# Genomic Characterization of the Novel *Aeromonas hydrophila* Phage Ahp1 Suggests the Derivation of a New Subgroup from phiKMV-Like Family

**DOI:** 10.1371/journal.pone.0162060

**Published:** 2016-09-07

**Authors:** Jian-Bin Wang, Nien-Tsung Lin, Yi-Hsiung Tseng, Shu-Fen Weng

**Affiliations:** 1 Institute of Medical Sciences, Tzu Chi University, Hualien 970, Taiwan; 2 Master Program in Microbiology and Immunology, School of Medicine, Tzu Chi University, Hualien 970, Taiwan; 3 Institute of Molecular Biology, National Chung Hsing University, Taichung 402, Taiwan; Centro Nacional de Biotecnologia (CNB-CSIC), SPAIN

## Abstract

*Aeromonas hydrophila* is an opportunistic pathogenic bacterium causing diseases in human and fish. The emergence of multidrug-resistant *A*. *hydrophila* isolates has been increasing in recent years. In this study, we have isolated a novel virulent podophage of *A*. *hydrophila*, designated as Ahp1, from waste water. Ahp1 has a rapid adsorption (96% adsorbed in 2 min), a latent period of 15 min, and a burst size of 112 PFU per infected cell. At least eighteen Ahp1 virion proteins were visualized in SDS-polyacrylamide gel electrophoresis, with a 36-kDa protein being the predicted major capsid protein. Genome analysis of Ahp1 revealed a linear doubled-stranded DNA genome of 42,167 bp with a G + C content of 58.8%. The genome encodes 46 putative open reading frames, 5 putative phage promoters, and 3 transcriptional terminators. Based on high degrees of similarity in overall genome organization and among most of the corresponding ORFs, as well as phylogenetic relatedness among their DNAP, RNAP and major capsid proteins, we propose a new subgroup, designated Ahp1-like subgroup. This subgroup contains Ahp1 and members previously belonging to phiKMV-like subgroup, phiAS7, phi80-18, GAP227, phiR8-01, and ISAO8. Since Ahp1 has a narrow host range, for effective phage therapy, different phages are needed for preparation of cocktails that are capable of killing the heterogeneous *A*. *hydrophila* strains.

## Introduction

*Aeromonas hydrophila*, a gram-negative, rod-shaped, non-spore-forming and facultatively anaerobic bacterium, is widely distributed in aquatic environments, drinking water, chlorinated water supply, and a wide range of food [1–3]. It causes various human infections such as bacteremia, pneumonia, endocarditis, empyema, arthritis, biliary tract infections, peritonitis, and skin and soft-tissue infections [4–8]. This species also causes diseases in fish, including *Aeromonas* septicemia, red sore disease and ulcerative infections mainly affecting carp and catfish [9]. The prevalence of *A*. *hydrophila* in Taiwan has been increasing; for example, among 129 patients with soft-tissue infections caused by *Aeromonas* species in Chi Mei Medical Center in Taiwan during 2009–2011, 77 (59.7%) were identified to be infected by *A*. *hydrophila* [10]. Although it has been demonstrated that third- and forth-generation cephalosporins and fluoroquinolones were effective against over 80% of the infections caused by *Aeromonas* species in Taiwan [8, 11, 12], the increasing rates of antibiotic resistances have raised the concern in treatment of *A*. *hydrophila* infections [13–16].

Bacteriophages are viruses specifically infecting their bacterial hosts and are estimated to be the most widely distributed and diverse entities in the biosphere. It has been suggested that the activities of bacteriophages are driven forces in maintaining genetic diversity amongst the bacterial community [17]. However, despite the importance of *A*. *hydrophila* in causing infections, only a few bacteriophages infecting this bacterium have been described, including characterization of myophages Aeh1, Aeh2, PM2, pAh1-C, pAh6-C, and VTCCBPA6, and filamentous phage PM3 [18–27], and sequencing of the myophage CC2 [28].

In this study, a lytic podophage infecting *A*. *hydrophila* was isolated from waste water, designated as Ahp1, and characterized. Analysis of nucleotide and amino acid sequences revealed that the Ahp1 genome has an organization similar to that of the phiKMV-like phages. However, phylogenetic analysis indicated that Ahp1 is most closely related to phages including *Aeromonas salmonicida* phage phiAS7, *Cronobacter sakazakii* phage GAP227, *Yersinia enterocolitica* phages phi80-18, phiR8-01, and ISAO8. Our analysis thus suggests the clustering of a new subgroup containing these phages, which were previously classified within the phiKMV-like subgroup. To our knowledge, this is the first characterized podophage infecting *A*. *hydrophila*.

## Materials and Methods

### Bacterial strains, phage and growth conditions

Bacterial strains used in this study are listed in Table 1. Luria Bertani (LB) broth and LB agar (Bacto) at 30°C were used to grow bacteria: *A*. *hydrophila* at 30°C, *Xanthomonas campestris* pv. campestris at 28°C, and the other strains at 37°C. Bacterial growth was monitored turbidimetrically by measuring optical density at 600 nm (OD_600_), in which an OD unit of 1.0 corresponded to 1.8 × 10^8^ CFU/ml. Newly isolated *A*. *hydrophila* strains were identified by 16S rDNA sequencing using specific primers [29].

**Table 1:** Phage and bacterial strains used in this study.

Strain(s)	Descriptions	Reference or source
*Aeromonas hydrophila* phage		
Ahp1	Environmental isolate	This study
*Aeromonas hydrophila*		
7966	ATCC type strain, Ap^r^	ATCC
43414	ATCC type strain, AP^r^	ATCC
AH19288	Clinical isolate from Buddhist Tzu Chi General Hospital, Ap^r^	This study
AH60114, AH300206	Clinical isolates from Hualien Armed Forces General Hospital, Ap^r^	This study
Hua-1, Hua-2	Sick fish isolates from Hualien Animal and Plant Disease Control Center, Ap^r^	This study
H1 to H35	Environmental isolates, Ap^r^	This study
*Acinetobacter baumannii*		
17978	ATCC type strain, Ap^r^	ATCC
*Escherichia coli*		
DH5α	F^- φ^80d*lac*ZΔM15Δ(*lacZYA*-*argF*) U169 *recA1 endA1 hsdR*17 (r_k_^−^, m_k_^+^) *pho*A *supE44* λ^−^ *thi*-*1 gyrA96 relA1*	[30]
*Klebsiella pneumonia*		
Kp-6	Clinical isolate, Ap^r^	N. T. Lin^a^
*Staphylococcus aureus*		
8325	NCTC type strain, Ap^r^	NCTC
*Vibrio parahaemolyticus*		
VP93	Clinical isolate, Ap^r^	M. S. Yu^b^
*Vibrio harveyi*		
BAA-1117	*lux*N::tn5Kan	ATCC
*Xanthomonas campestris* pv. campestris		
P20H	Nonmucoid mutant, Ap^r^	Y. H. Tseng

^*a*^N. T. Lin, Master Program in Microbiology and Immunology, School of Medicine, Tzu Chi University, Hualien, Taiwan.

^*b*^M. S. Yu, Master Program in Microbiology and Immunology, School of Medicine, Tzu Chi University, Hualien, Taiwan.

### Phage isolation and test for host range

The procedures described previously [31] were used for phage isolation, plaque assay and spot test. To test for host range, spot test was performed by including the bacterial strains separately in the double-layered agar plates and 5 μl of phage lysates (10^7^ PFU) were spotted onto the bacterial lawns and dried in a laminar flow for 10 min and incubated for 16-18h. The experiments were repeated 3 times.

### Adsorption test

Cells of *A*. *hydrophila* ATCC 7966 (0.6 U of OD_600_) in LB medium were infected with Ahp1 to give a multiplicity of infection (MOI) of 0.0001 and incubated at 30°C. Aliquots of 100 μl were taken at 2-min intervals (up to 17 min), diluted in 0.9 ml of cold LB, and centrifuged (12,000 × *g*, 5 min). The unadsorbed phages in supernatants were assayed.

### One-step growth

Cells of *A*. *hydrophila* ATCC 7966 (0.6 U of OD_600_) were harvested by centrifugation and resuspended in 0.9 ml of fresh LB medium (ca. 10^9^ CFU/ml). Ahp1 was added at an MOI of 0.0001 and allowed to adsorb for 30 min at 4°C. The mixture was centrifuged at 12,000 × *g* for 10 min. The pellets containing infected cells were resuspended in 50 ml of LB and incubated at 30°C. Samples were taken at 5-min intervals (up to 35 min), immediately centrifuged at 12,000 × *g* for 2 min, then the supernatants were diluted in cold LB medium, followed by determining the phage titers.

### Purification of phage particles

Phage lysates (200 ml, ca. 1.0 × 10^10^ PFU/ml) were centrifuged at 7,800 × g for 10 min. The supernatants were passed through a 0.45-μm-pore-size membrane filter and centrifuged at 22,000 rpm (BECKMAN COULTER Avanti-J25I) for 2 h at 4°C. The pellets were suspended in 1.0 ml of SM buffer (0.05 M Tris-HCl, pH 7.5 containing 0.1 M NaCl, 0.008 M MgSO_4_‧7H_2_O, and 0.01% gelatin) and loaded on block gradient of CsCl (ρ = 1.50, 1.48, 1.45, 1.43, and 1.40 g/cm^3^), followed by ultracentrifugation at 30,000 rpm for 3 h at 4°C with the SW41Ti rotor in a BECKMAN Optima LE-80K Ultracentrifuge. The banded phage particles were recovered, desalted with Amicon Ultra Centrifugal Filters (10,000 MWCO, Millipore Corporation, Ireland), and then stored at 4°C until used.

### DNA techniques

The procedures described previously [31] were used for isolation and restriction enzyme digestion of the phage DNA. Pulsed-field gel electrophoresis (PFGE) was performed as described previously [32], by using a CHEFDRIII System (Bio-Rad Laboratories, Hercules, CA, USA) at 6 V/cm with pulse ramps from 3.5 to 4s for 19.5h for the intact genomic DNA at 9°C in 0.5 × Tris-borate-EDTA buffer, pH 8.0. Midrange I PFG Markers (New England Biolabs) were used as molecular size standards.

### Electron microscopy

To observe the phage morphology, 10 μl of Ahp1 suspension (1.0 × 10^12^ PFU/ml) was dropped onto the surface of a formvar-coated grid (400 mesh copper grids), let stand for 3 min, stained with 2% uranyl-acetate for 30s, and examined in a Hitachi H-7500 transmission electron microscope operated at 80 kV.

### Whole genome sequencing and *in silico* analysis

The genomic DNA of Ahp1 was sequenced by using Next Generation Sequencing system (Illumina Solexa technology) with end paired method.

The genome of Ahp1 was scanned for potential open reading frames (ORFs) with ORF Finder (http://www.ncbi.nlm.nih.gov/projects/gorf/), and GeneMarkS software [33]. Annotation was carried out by comparing translated ORFs in BLASTP (http://blast.ncbi.nlm.nih.gov/Blast.cgi). The presence of transmembrane domains was verified with TMHMM software [34]. Prokaryotic promoter regions were identified by using the BPROM prediction program on the SoftBerry website (http://www.softberry.com/). Potential phage promoter sites were scanned for using PHIRE software [35]. Palindromic repeat regions were identified by FindTerm program on the SoftBerry website. Putative terminators were defined as palindromic sequences followed by a U-rich stretch and a stable secondary structure (ΔG < −10 kcal/mol). ClustalW was used for multiple alignment which was performed with Molecular Evolutionary Genetics Analysis (MEGA) software 6.0.6 aided by manual adjustments [36]. Phylogenetic analysis was also performed with MEGA by using the neighbor-joining method with 1,000 bootstrap replicates.

### Nucleotide sequence accession number

The genome sequence of the *Aeromonas hydrophila* phage Ahp1 has been deposited in GenBank under accession number KT949345.

## Results and Discussion

### Isolation and general properties of Ahp1

Thirteen water samples, including those from sewages, wastewater, and aquariums were screened separately by spot tests on the lawns of four *A*. *hydrophila* strains, including ATCC 7966 and three clinical isolates (AH19288, AH60114, and AH300206). One phage was isolated and designated as Ahp1.

**Fig 1:**
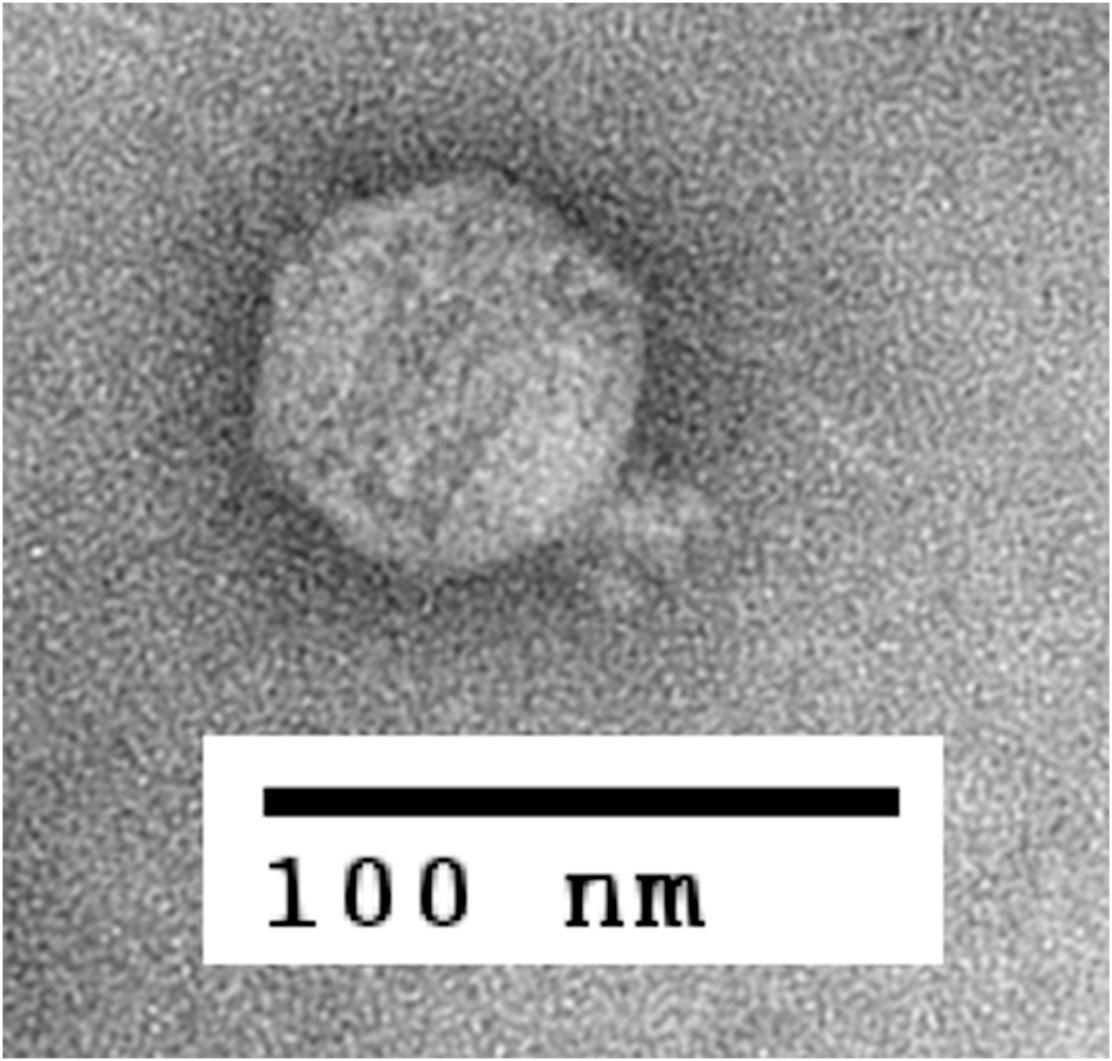
Transmission electron micrograph of *A*. *hydrophila* phage Ahp1. Ahp1 was negatively stained with 2% uranyl-acetate. The bar corresponds to 100 nm.

To obtain high titer lysate, different conditions were tested. Results showed that infecting a culture of *A*. *hydrophila* ATCC 7966 (200 ml of LB medium in a 500 ml flask) at exponential phase (0.8 unit of OD_600_) with an MOI of 0.0001 caused a complete lysis of the culture within 150 min, resulting in the production of approximately 2.5 × 10^10^ PFU/ml of phage progeny. Transmission electron microscopy revealed that Ahp1 possessed an icosahedral head (62 nm in diameter) and a short tail (12.5 nm in length). The morphology was thus similar to a typical member of *Podoviridae* family (Fig 1). Since no podophage of *A*. *hydrophila* has been reported, Ahp1 appears to be the first member of *Podoviridae* infecting this bacterium.

It has been shown that several lipid-containing phages, such as PRD1, PM2, mycobacteriophage D29 and DS6A, are inactivated by chloroform [37–40]. In this study, about 10^8^ PFU of the phage suspension (100 μl) was mixed with chloroform at concentrations from 1 to 5%, shaken for 5 min, followed by incubation of the mixture at room temperature for 25 min. Results showed that at 5%of chloroform, no infective particle was detectable, indicating that Ahp1 is sensitive to chloroform and suggesting that it may contain structural lipids.

The adsorption rate of Ahp1 on *A*. *hydrophila* ATCC 7966 is shown in S1 Fig. Approximately 96% of Ahp1 was adsorbed to the host cells within 2 min and no free phages were detectable in the supernatant at 4 min in our assay conditions, indicating a highly efficient adsorption. To understand the growth, one-step growth curve of Ahp1 on *A*. *hydrophila* ATCC 7966 was determined. As shown in S2 Fig, Ahp1 exhibited a latent period of about 15 min, and a short growth period of about 25 min. The average burst size was estimated to be 112 PFU per infected cell.

### Ahp1 has a narrow host range

To test for host range, lawns of 42 *A*. *hydrophila* strains listed in Table 1 were subjected to spot tests with Ahp1. Results showed that only 6 (14.3%, including ATCC 7966, H6, H10, H23, H30 and H32) strains displayed clearing zones, and the others were resistant to Ahp1. All the susceptible *A*. *hydrophila* strains, except ATCC 7966, were environmental isolates.

Bacterial strains belonging to 7 species other than *A*. *hydrophila* (Table 1), *Acinetobacter baumannii*, *Escherichia coli*, *Klebsiella pneumoniae*, *Staphylococcus aureus*, *Vibrio parahaemolyticus*, *Vibrio harveyi*, and *Xanthomonas campestris* pv. campestris were also subjected to spot test. Results showed that none of these bacteria were susceptible to Ahp1. These results indicated that Ahp1 has a narrow host range and more phages are needed to form a cocktail for future therapeutic use.

### Ahp1 has a buoyant density between 1.48 and 1.45 g/cm^3^

During phage purification, Ahp1 lysates (ca. 1.0 × 10^12^ PFU) were subjected to ultracentrifugation in a discontinuous CsCl gradient (ρ = 1.50, 1.48, 1.45, 1.43, and 1.40 g/cm^3^). Ahp1 was found to band above the 1.48 g/cm^3^ block, suggesting that Ahp1 has a buoyant density between 1.48 and 1.45 g/cm^3^.

### The Ahp1 genome is about 42 kb in size

**Fig 2:**
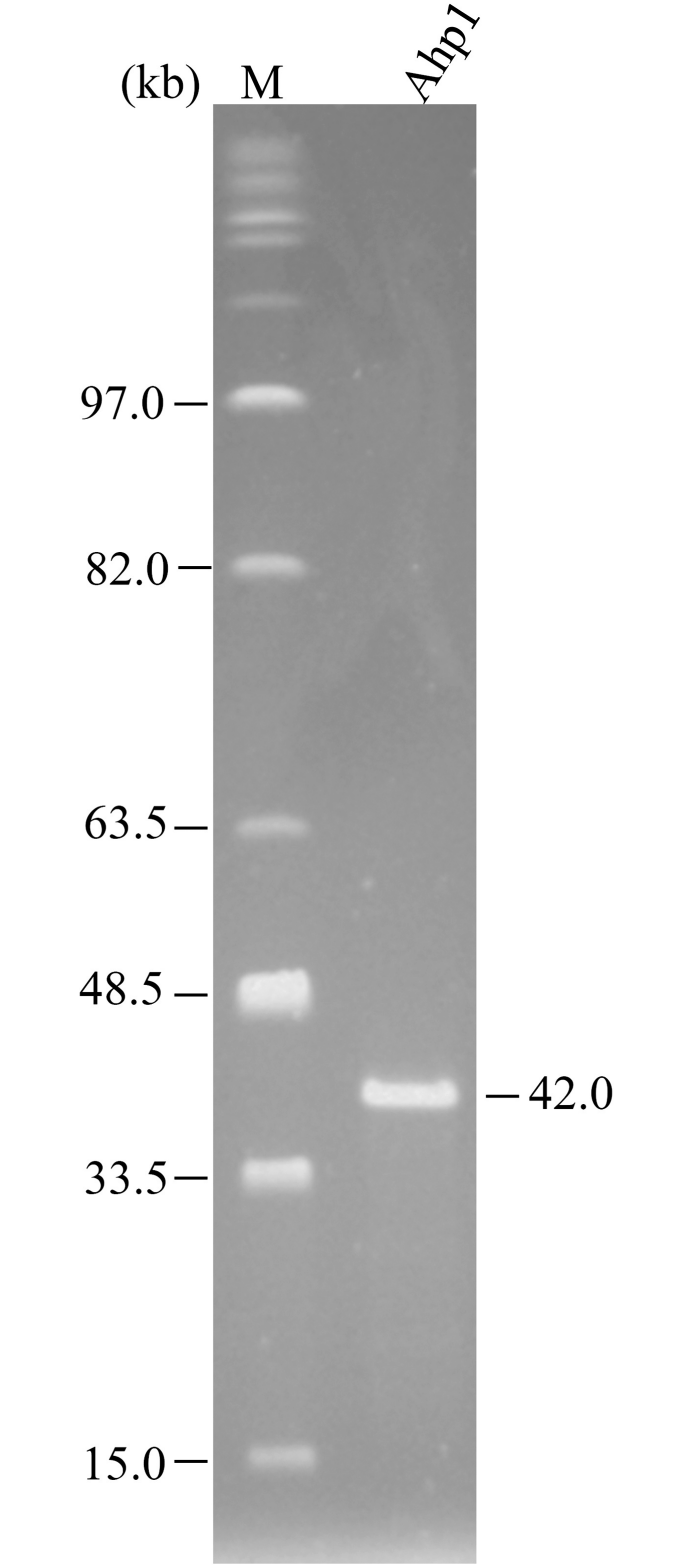
Estimation of genome size of phage Ahp1 by pulsed-field gel electrophoresis. Lanes: M, midrange I PFG markers; Ahp1, genomic DNA of Ahp1.

Several restriction endonucleases were tested and the Ahp1 DNA was found to be cut by EcoRV, HindIII, and EcoRI into 2, 5, and 4 fragments, respectively (data not shown). Digestibility by type II restriction enzymes suggests that Ahp1 has a double-stranded DNA genome. To estimate the Ahp1 genome size, DNA from phage particles was subjected to PFGE. As shown in Fig 2, the genome size of Ahp1 was estimated to be 42 kb, similar to the value estimated by summing up the fragment sizes obtained from restriction digests.

### The Ahp1 virion consists of at least 18 proteins

**Fig 3:**
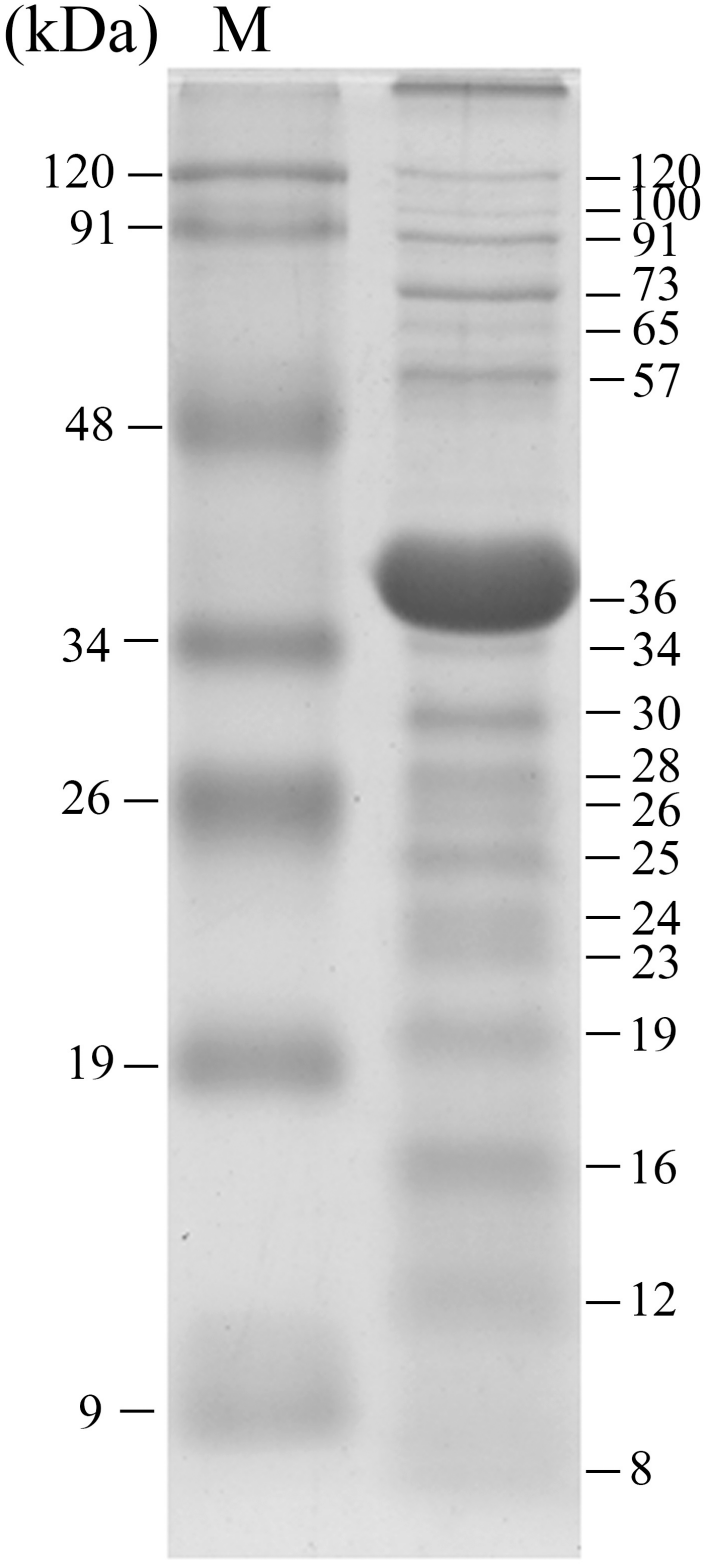
SDS-polyacrylamide gel (8–16% gradient) electrophoresis (SDS-PAGE) of Ahp1 virion proteins. About 5 × 10^11^ PFU of purified phage particles were boiled in sample buffer (100 mM Tris-HCl pH 6.8, 4% SDS, 0.2% bromophenol blue, 20% glycerol, 200 mM dithiothreitol) (20 μl) and loaded onto the well. Lane M, prestained middle range protein markers (Protech Technology). Estimated molecular masses are indicated to the right.

To analyze the virion proteins, purified Ahp1 phage particles were subjected to precast 8–16% gradient polyacrylamide gel (Bio-Rad Laboratories, Hercules, CA, USA, CAT#456–1103) separation following the procedures described previously [31]. As shown in Fig 3, at least 18 protein bands were visualized upon staining the gel with Coomassie brilliant blue. The band with an apparent molecular mass of 36 kDa was the most abundant protein, suggesting that it is the major coat protein of Ahp1.

### Sequencing of the Ahp1 genome

**Fig 4:**
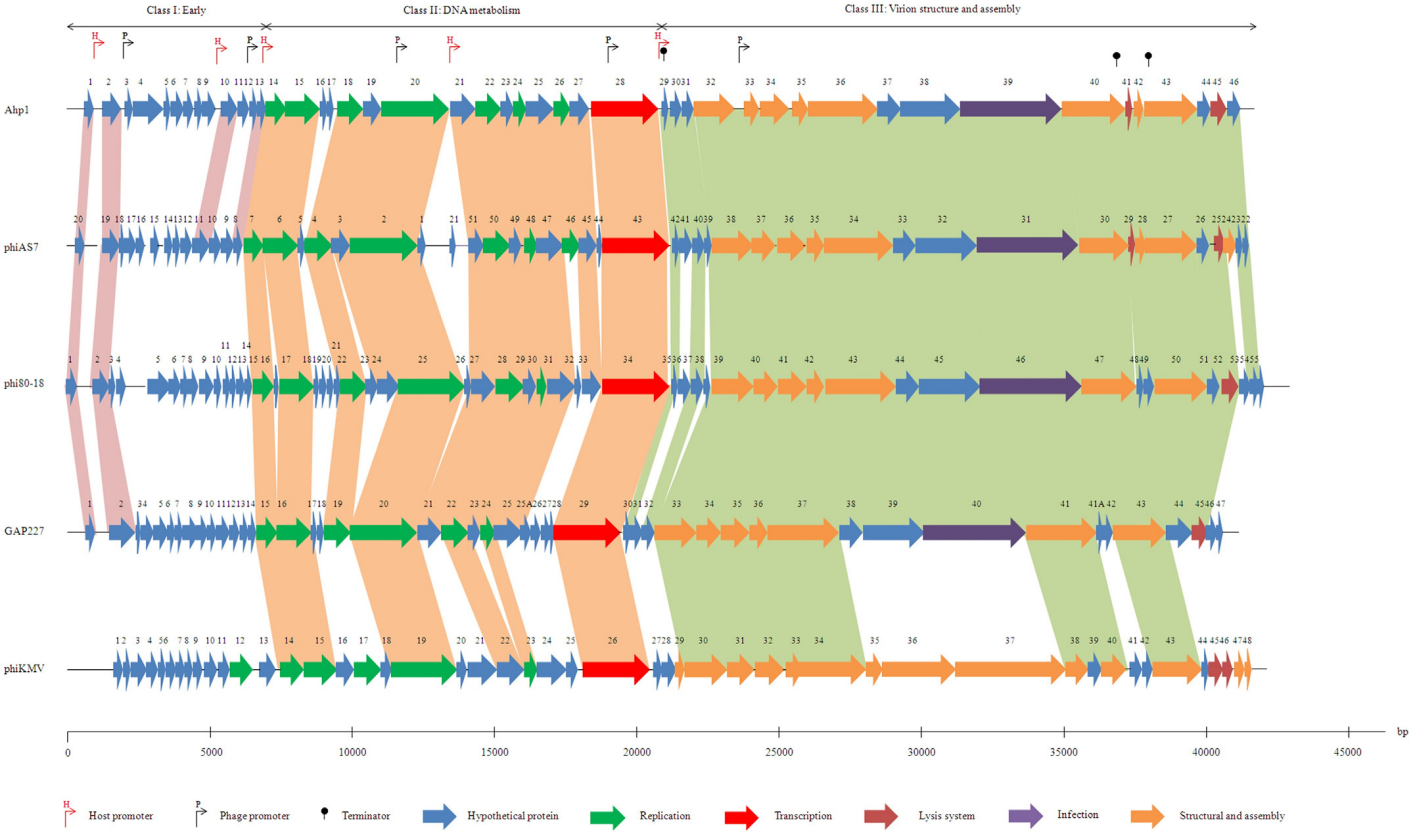
Genome organization of Ahp1 and similar phages. Predicted ORFs are numbered for Ahp1 and other members. The ruler below represents the features of the genome. PhiAS7, phage of *Aeromonas salmonicida*; phi80-18, phage of *Yersinia enterocolitica*; GAP227, phage of *Cronobacter sakazakii*, phiKMV, phage of *Pseudomonas aeruginosa*. Three closely related *Yersinia enterocolitica* phages ISAO8, phi80-18, and phiR8-01 have been available. Shown here is only phi80-18.

The genomic DNA of Ahp1 was extracted from CsCl-purified particles and sequenced by next generation sequencing and primer walking. Results showed that the Ahp1 genome consisted of 42,167 bp, in good agreement with that estimated in PFGE (Fig 2). It had a G + C content of 58.8%, slightly lower than that of the host genome (61.5%). Open reading frame (*orf*s) prediction suggested 46 *orf*s, which occupied 92.4% of the genome. These *orf*s, all located on the same strand, were transcribed in the same direction (Fig 4). All *orf*s began with AUG, except *orf18* and *22* which used GUG and *orf35* and *42* which used UUG as the initiation codon (Table 2). Five bacterial promoters (red bent arrows), five phage promoters (black bent arrows), and three factor-independent terminators (black knobs) were predicted (Table 3, Fig 4).

**Table 2:** *Aeromonas hydrophila* phage Ahp1 genomic DNA.

*orf*	Start	Stop	G+C (%)	Length (aa)	Mass (kDa)	Identity	Accession number	Related proteins
01	683 (ATG)	958 (TGA)	56.5	91	10.3	56/84 (67%)	YP_007007792.1	Hypothetical protein, phiAS7_00020 (*Aeromonas* phage phiAS7)
02	1315 (ATG)	1929 (TGA)	59.3	204	22.4	101/160 (63%)	YP_007007791.1	Hypothetical protein, phiAS7_00019 (*Aeromonas* phage phiAS7)
03	2092 (ATG)	2325 (TAA)	61.1	77	8.9			No similarity
04	2368 (ATG)	3456 (TAA)	59.6	362	40.2			No similarity
05	3460 (ATG)	3660 (TGA)	54.7	66	7.2			No similarity
06	3657 (ATG)	4082 (TGA)	61.0	141	15.2	21/68 (31%)	WP_047663885.1	Helicase (*Raoultella planticola*)
07	4079 (ATG)	4387 (TGA)	59.9	102	11.3			No similarity
08	4449 (ATG)	4679 (TGA)	59.3	76	8.7	26/50 (52%)	CCI88402.1	Hypothetical protein, BN110_021 (*Yersinia* phage phiR8-01)
09	4676 (ATG)	5104 (TAG)	58.5	142	16.2	18/55 (33%)	WP_023986013.1	D-amino-acid dehydrogenase (*Mycobacterium*)
10	5348 (ATG)	5908 (TGA)	61.1	186	21.0	34/74 (46%)	YP_007007783.1	Hypothetical protein, phiAS7_00011 (*Aeromonas* phage phiAS7)
11	5908 (ATG)	6282 (TAA)	60.8	124	13.1	17/29 (59%)	WP_055395407.1	Hypothetical protein (*Acidovorax* sp. SD340)
12	6293 (ATG)	6520 (TGA)	61.0	75	8.4			No similarity
13	6517 (ATG)	6780 (TAA)	51.9	87	9.8	33/76 (43%)	YP_007007780.1	Hypothetical protein, phiAS7_00008 (*Aeromonas* phage phiAS7)
14	6897 (ATG)	7592 (TAG)	60.1	231	25.9	105/226 (46%)	AKQ07708.1	DNA primase (*Yersinia* phage vB_YenP_ISAO8)
15	7579 (ATG)	8829 (TGA)	59.2	416	46.3	303/414 (73%)	AKQ07709.1	DNA helicase (*Yersinia* phage vB_YenP_ISAO8)
16	8838 (ATG)	9047 (TAA)	59.0	69	7.7	17/54 (31%)	WP_047676134.1	Glyoxalase (*Paenibacillus chondroitinus*)
17	9040 (ATG)	9222 (TAA)	56.8	60	6.6			No similarity
18	9294 (GTG)	10199 (TGA)	60.4	301	34.2	130/305 (43%)	YP_007007776.1	Putative ATP-dependent DNA ligase, phiAS7_00004 (*Aeromonas* phage phiAS7)
19	10210 (ATG)	10827 (TAA)	59.1	205	23.3	47/131 (36%)	YP_007236327.1	Putative nucleotidyltransferase, BN109_024 (*Yersinia* phage phi80-18)
20	10827 (ATG)	13313 (TAA)	59.7	828	93.9	583/829 (70%)	AKQ07710.1	DNA polymerase (*Yersinia* phage vB_YenP_ISAO8)
21	13329 (ATG)	14204 (TGA)	62.7	291	31.6	157/243 (65%)	CCI88414.1	37L, BN110_033 (*Yersinia* phage phiR8-01)
22	14201 (GTG)	15145 (TAA)	57.6	314	35.4	203/303 (67%)	CCI88415.1	Hypothetical protein, BN110_034 (*Yersinia* phage phiR8-01)
23	15132 (ATG)	15557 (TAA)	61.3	141	15.0	46/115 (40%)	AKQ07687.1	Hypothetical protein (*Yersinia* phage vB_YenP_ISAO8)
24	15550 (ATG)	15966 (TGA)	60.9	138	15.2	97/140 (69%)	AKQ07688.1	DNA endonuclease (*Yersinia* phage vB_YenP_ISAO8)
25	15963 (ATG)	16937 (TGA)	61.9	324	36.5	209/325 (64%)	YP_007007819.1	Hypothetical protein, phiAS7_00047 (*Aeromonas* phage phiAS7)
26	16934 (ATG)	17485 (TGA)	60.0	183	20.9	100/166 (60%)	YP_007007818.1	Putative kinase phosphatase, PhiAS7_00046 (*Aeromonas* phage phiAS7)
27	17482 (ATG)	18126 (TGA)	62.5	214	24.1	90/212 (42%)	CCI88419.1	Hypothetical protein, BN110_038 (*Yersinia* phage phiR8-01)
28	18240 (ATG)	20687 (TAA)	58.9	815	92.3	423/818 (52%)	AKQ07690.1	RNA polymerase (*Yersinia* phage vB_YenP_ISAO8)
29	20846 (ATG)	21028 (TAA)	52.5	60	6.6	30/48 (63%)	AKQ07691.1	Hypothetical protein (*Yersinia* phage vB_YenP_ISAO8)
30	21116 (ATG)	21475 (TAA)	58.9	119	13.7	19/45 (42%)	XP_004926689.1	Uncharacterized protein, LOC101744261 (*Bombyx mori*)
31	21475 (ATG)	21867 (TAA)	60.8	130	13.8	71/130 (55%)	YP_007007812.1	Hypothetical protein, phiAS7_00040 (*Aeromonas* phage phiAS7)
32	21898 (ATG)	23379 (TGA)	60.4	493	55.7	284/477 (60%)	YP_007236342.1	Head portal-like protein, BN109_039 (*Yersinia* phage phi80-18)
33	23766 (ATG)	24272 (TGA)	60.7	168	17.7	90/170 (53%)	YP_007007809.1	Putative scaffolding protein, phiAS7_00037 (*Aeromonas* phage phiAS7)
34	24337 (ATG)	25362 (TAA)	59.0	341	36.9	249/336 (74%)	YP_007007808.1	Putative major capsid protein, phiAS7_00036 (*Aeromonas* phage phiAS7)
35	25451 (TTG)	26026 (TAA)	57.5	191	21.6	92/191 (48%)	YP_007007807.1	Putative tail tubular A protein, phiAS7_00035 (*Aeromonas* phage phiAS7)
36	26029 (ATG)	28581 (TAA)	57.7	850	94.5	466/854 (55%)	YP_007007806.1	Putative tail tubular B protein, phiAS7_00034 (*Aeromonas* phage phiAS7)
37	28581 (ATG)	29375 (TAA)	59.9	264	28.0	114/252 (45%)	YP_007007805.1	Hypothetical protein, phiAS7_00033 (*Aeromonas* phage phiAS7)
38	29375 (ATG)	31549 (TAA)	60.6	724	78.5	271/711 (38%)	YP_007007804.1	Hypothetical protein, phiAS7_00032 (*Aeromonas* phage phiAS7)
39	31553 (ATG)	35311 (TAA)	60.3	1252	134.4	568/1259 (45%)	CCI88385.1	Lytic transglycosylase, catalytic, BN110_004 (*Yersinia* phage phiR8-01)
40	35376 (ATG)	37715 (TAG)	49.9	779	82.4	65/128 (51%)	AKQ07702.1	Tail fiber protein (*Yersinia* phage vB_YenP_ISAO8)
41	37724 (ATG)	37906 (TGA)	51.9	60	6.5	37/61 (61%)	YP_009223416.1	Type II holin (*Cronobacter* phage Dev-CD-23823)
42	37884 (TTG)	38249 (TAG)	60.1	121	13.2	64/99 (65%)	CCI88389.1	Hypothetical protein, BN110_008 (*Yersinia* phage phiR8-01)
43	38258 (ATG)	40183 (TAA)	59.1	641	71.8	478/641 (75%)	AKQ07715.1	DNA packaging protein (*Yersinia* phage vB_YenP_ISAO8)
44	40183 (ATG)	40605 (TAA)	64.1	140	14.7	45/127 (35%)	YP_007007798.1	Hypothetical protein, phiAS7_00026 (*Aeromonas* phage phiAS7)
45	40615 (ATG)	41157 (TAG)	60.6	180	19.8	117/172 (68%)	CCI88392.1	Prophage lysozyme, phage lysin, BN110_011 (*Yersinia* phage phiR8-01)
46	41212 (ATG)	41625 (TAA)	58.7	137	15.4	24/56 (43%)	YP_007007795.1	Hypothetical protein, phiAS7_00023 (*Aeromonas* phage phiAS7)

**Table 3:** Regulatory elements in the genome of Ahp1.

Position	*orf*^a^	Regulatory element sequence^b^
*E*. *coli* σ^70^-like promotors		
980_1203	2	TAGCGGTTGACAGGCTCGATAATCCCTGATATGATGGGCACCAT
5294_5337	10	TACCCGCTGACGTCCCTAAACGGACGCATTACCATCACCAAGTA
6721_6764	14	GCGTGGTTGAACGACCTGCTGGACAAGGGTGTCATCACCTACGA
13276_13319	21	AGTATCTGGCAAGCAAGAAACTCGAAGTTAAATTCTAAAGAGAG
20708_20751	29	AATAACTAGATAATACCTAGTTTAATACCTAGTTATAATCCTTA
Consensus promoter, *E*. *coli*		-------------TTGACA (-35)------------------TATAAT (-10)---------------
Phage promotors		
2021_2040	3	TCCCTGCACTCGCAGAGGGT
6242_6261	12	TGGATGCACTCGCAGATGAT
11515_11534	21	TGGCTGCACTTGCAGAGGAA
18810_18829	29	CTGCTGCACTCGCAGATGGT
22424_22443	33	TGGCTGCACTTGCAGATGGT
23591_23610	33	GGCTTCCAAGAGGGCCGAAG
ρ-independent terminators		
20963_21001	30	ATGGCCTTCCAGCTGGGCTGCCAGAAGGTTTTATTTCTC
36635_36689	41	AAGTCAATGCACTGTGTTTGCAACCGACACAGTGCGGGTTGGTGTTTATGCTAGG
38337_38370	44	AGCTGGAGGGCGAGTCCCTCCAGTTCCTTTCTGA

^*a*^ Number of the *orf* downstream of the element.

^*b*^ Underlined sequences represent the −35 and −10 boxes for the promoters and the palindromic sequence for the terminators.

### Genome organization

Members of phiKMV-like phages include *Pseudomonas aeruginosa* phage phiKMV and at least 13 related phages as available from data base. The genome of phiKMV and the related phages are divided into three classes: class I contains early genes, class II encodes proteins that participate in DNA metabolism, and class III contains genes required for virion structure, host lysis, and phage assembly. As shown in Fig 4, organization of the Ahp1 genome was similar to that of phiKMV and the related phages.

ORFs of Ahp1 shared high degrees of similarity with the homologs from three of the phiKMV-like phages including phiAS7, GAP227, and phi80-18 (Table 2, Fig 4), while lower degrees of similarity were shared with those from the other phiKMV-like phages. With number of the similar ORFs and range of % similarity in the parenthesis, they are phiAS7 (38/51 means that 38 of the 51 phiAS7 ORFs are similar, 33%-74%), GAP227 (28/47, 34%-73%), phi80-18 (31/55, 31%-70%), and phiKMV (14/47, 24%-39%). In addition, among the class I ORFs, only ORF1 and ORF2 were similar to the hypothetical proteins from phiAS7, phi80-18 and GAP227, and only ORF10 similar to phiAS7 ORF11 (Fig 4). In other words, more Ahp1 homologs are found in three of phiKMV-like phages phiAS7, phi80-18, and GAP227 than in the other phiKMV-related phages (NC_005045.1, NC_015585.1, NC_019454.1, NC_009936.1, NC_009935.1, NC_013649.2, NC_012662.1, NC_028675.1, NC_028850.1, and HE956707.1). Also, higher degrees of similarity are shared with the homologs from the three phiKMV-like phages than that from the other phiKMV-related phages (Table 2, Fig 4). These data suggest that Ahp1 is more closely related to phages phiAS7, phi80-18, and GAP227 than to the other phiKMV-related phages, suggesting that the phiKMV-related phages can be further divided into at least two subgroups.

It was also noted that in spite of the high degrees of similarity being shared between the homologous ORFs, organization of the phiAS7 genome was different from that of the other similar phages, with its ORF1-ORF20 and ORF22-ORF51 being inverted (S3 Fig). However, when the phiAS7 genome was redrawn by inverting both ORF1-ORF20 and ORF22-ORF51 regions, its gene order became largely the same as that of the other four phages (Fig 4). Our finding indicates that procedures for assembly of the phiAS7 contigs may need to be revisited.

### Gene products of Ahp1

Protein products encoded by the Ahp1 class I *orf*s were either hypothetical or sharing no similarity to those in database (Table 2), similar to the cases in phiKMV-like phages. Roucourt et al. suggest, through yeast two-hybrid experiments [41], that class I genes of phiKMV although most being hypothetical have roles in bacteriophage-host interaction. However, it would be difficult to assign common functions for these Ahp1 ORFs, since they are highly varied in amino acid sequences.

*Orf14*, the first gene in class II region encoded a potential DnaG-like primase with a PHA02031 (N-terminal) domain (aa 13–69) conserved among phage DNA primase. The protein product of *orf15* has domains similar to those of a DNA helicase, which unwinds the DNA duplex during replication initiation: one at aa 188–195 (AXXXXGKT) similar to the phosphate-binding loop (GXXXXGK-T/S) [42] and one at aa 293–298 (IVVFDM) similar to a Mg^2+^-binding site (hhhhDE, where h is a hydrophobic residue) [43]; these domains are also known as Walker A and B motifs, respectively. *Orf*16 encoded a hypothetical protein, while protein encoded by *orf17* found no similar proteins in database. ORF18 was identified as a potential ATP-dependent DNA ligase, with a DNA_ligase_A_M domain (aa 1–205, pfam01068) which included i) an active site motif (aa 6–11, KRDEFR corresponding to K-Y/A-D-G-X-R) consistently present in ATP-dependent DNA ligases [44] and ii) critical residues (K203 and K205 corresponding to K238 and K240 of phage T7 ligase, respectively) responsible for catalysis and nick recognition [45]. ORF19 was identified as a potential nucleotidyltransferase, containing a NT_ClassII_CCAase domain (aa 23–55, cd05398), which is a CCA-adding enzyme, adding the sequence cytidine(C)-cytidine-adenosine (A) one nucleotide at a time to the 3’ end of tRNA in a template-independent reaction [46]. ORF20, a potential DNA polymerase (DNAP), possessed a DNA_polA domain (aa 377–780, pfam00476). *Orf21* encoded a hypothetical protein. ORF22, a potential 5’-3’ exonuclease, possessed an active site of PIN_53EXO domain (aa 90–156, cd09859) that is conserved in bacterial DNA polymerase [47]; within the active site was a set of conserved acidic residues (E130, D132, D133, D152, and D154) similar to that essential for binding divalent metal ions required for nuclease activity in *Taq* DNA polymerase (DNAP) [48]. *Orf23* encoded a hypothetical protein. *Orf24*, encoding a potential endonuclease, contained an Endonuclease_7 domain (aa 23–93, pfam02945). *Orf25* encoded a hypothetical protein. *Orf26* encoded a potential polynucleotide 5’ kinase/3’phosphatase, containing an acid_phosphat_B domain (aa 20–147, pfam03767). *Orf27*, containing an ADK domain (aa 5–30, cd01428), encoded a potential ATP-binding protein.

**Fig 5:**
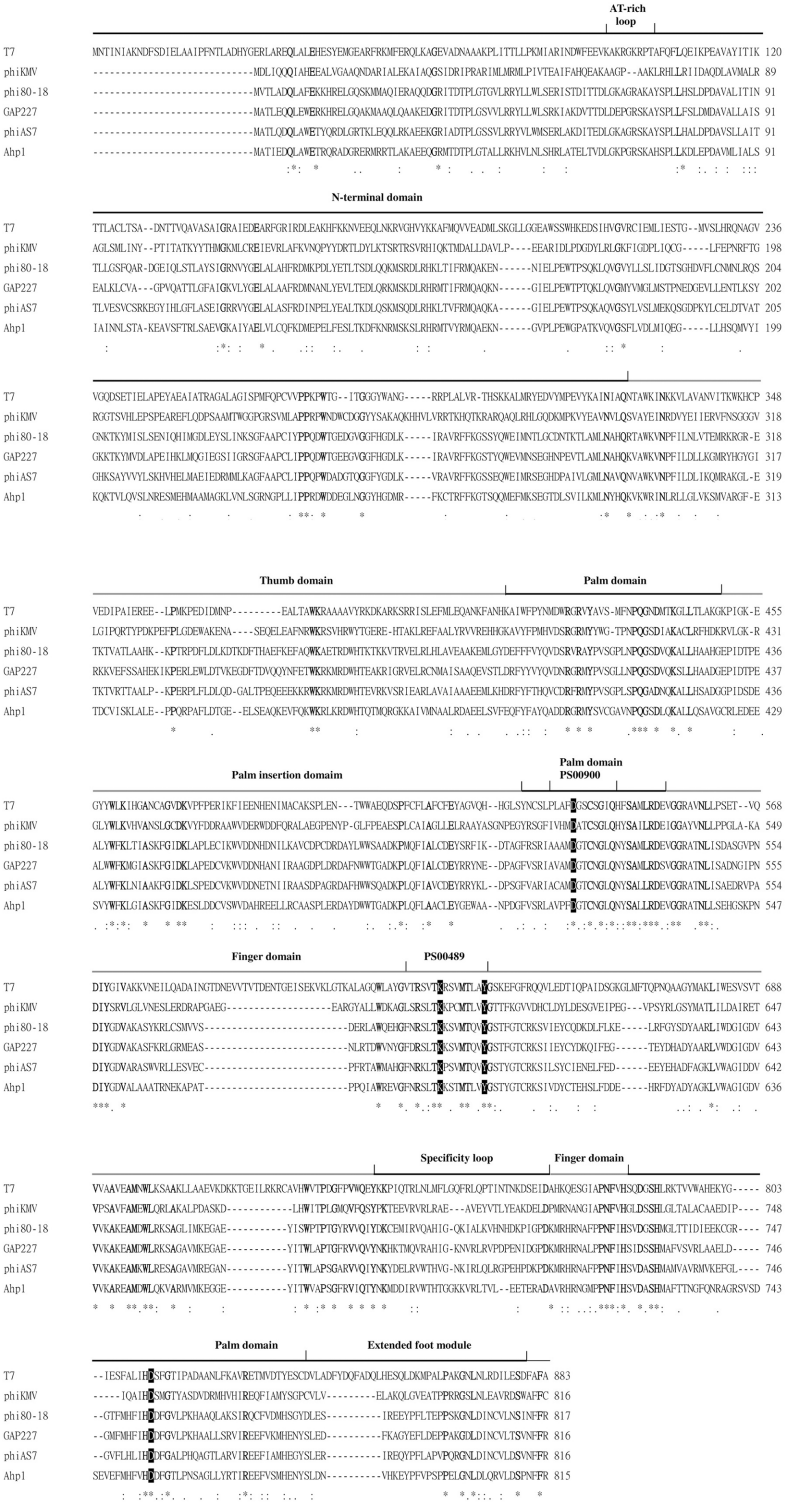
Sequence alignment of RNA polymerase (RNAP) from T7, phiKMV, phi80-18, GAP227, phiAS7, and Ahp1 by ClustalW. Lines superposed over the alignment show the major features obtained experimentally for T7 RNAP. Black shadowed residues indicate functionally important residues in T7 RNAP. Boldface residues are highly conserved amino acids within known RNAP. Symbols: “*”, identical residues in all sequences, “:”, highly conserved residues, “.”, weakly conserved residues.

The last gene in class II region was *orf28* encoding a potential DNA-dependent RNA polymerase (RNAP) as mentioned above. Alignment of the Ahp1 DNA-dependent RNAP with that of other phages including T7, phiKMV, phi80-18, GAP227 and phiAS7 is shown in Fig 5. Active sites PS00900 (P-[LIVM]-XX-D-[GA]-[ST]-[AC]-[SN]-[GA]-[LIVMFY]-Q) and PS00489 ([LIVMF]-X-R-XXX-K-XX-[LIVMF]-M-[PT]-XX-Y) [49–51] are conserved in bacteriophage-type RNAP. The invariant D537 (Palm domain) and D812 (Palm domain) in T7 RNAP, the catalytically most critical residues that directly involved in phosphodiester bond formation by coordinating Mg^2+^ ions [51, 52], are also conserved in Ahp1 (D514 and D754). The conserved K631 and Y639 in the Finger domain of T7 RNAP [50, 51], that are important catalytic residues of the active site, were also found in Ahp1 RNAP (K584 and Y592). However, the AT-rich recognition loop and the specificity loop that interact with T7 promoters [53, 54] were not conserved in the RNAP of Ahp1 and phiKMV-like phages (Fig 5).

Class III region contained genes potentially involved in virion structure and assembly, except *orf*s *29*, *30*, *31*, *37*, *38*, *44*, and *46* that encoded hypothetical proteins. *Orf32*, encoded a potential head portal protein, containing a Head-tail_con domain (aa 2–453, pfam12236) found in bacteria and phages. ORF33 showed 31% similarity to the scaffolding protein of *Burkholderia* phage JG068 (YP_008853872.1). Amino acid position 11–337 of ORF34 exhibited similarities to domain PHA02004 conserved in major capsid proteins of *Pseudomonas* phage Bf7 (YP_005098192.1), and *Burkholderia* phage JG068 (YP_008853873.1). In addition, ORF34 had a calculated molecular mass of 36.9 kDa, similar to that observed for the most abundant band, thought to be the major coat protein, in the SDS-PAGE separation of the Ahp1 virion proteins (Fig 3, Table 2). Amino acid position 2–183 of ORF35 was identified as a potential tail tube protein, which showed similarity to domain PHA00428 conserved in tail tube protein A of *Pseudomonas* phage Bf7 (YP_005098193.1), and *Burkholderia* phage JG068 (YP_008853874.1). ORF36 was identified as a potential tail tube protein B which was similar to that of *Ralstonia* phage RSB1 (YP_002213723.1), and *Burkholderia* phage JG068 (YP_008853875.1). Notably, such a gene order (head portal protein-scaffolding protein-major capsid protein-tail tubular protein A and B) is also observed in all phiKMV-like phages [55]. ORF39, similar to lytic transglycosylase which has a LT_GEWL domain (cd00254) that contains an invariant Glu (E30) [56] for catalysis and a conserved Tyr (Y105) [57], had the LT_GEWL domain being located in aa 30–106 which contained E28 and Y103. ORF40, containing a region (aa 19–127) resembling the Phage_T7_tail domain (pfam03906), was a potential tail fiber protein. *Orf41* was identified as a putative holin, because it was small in size with a transmembrane domain, located near the predicted endolysin gene (*orf45*) [58]; in addition, no other possible protein similar to holin in database was found. ORF42 and ORF43 were predicted to be DNA maturase A and B, respectively, based on their similarity to that of phiAS7 (YP_007007800.1) and *Burkholderia* phage JG068 (YP_008853883.1). ORF45, containing a region (aa 30–170) similar to the endolysin_autolysin domain (cd00737) was annotated to be the endolysin of Ahp1.

Many phages of Gram-negative bacteria encode internally overlapping Rz/Rz1 proteins, with the genes situating immediately downstream of the endolysin gene, to enhance bacterial lysis when the outer membrane is stabilized by divalent cations [59, 60]. However, no similar proteins were found in Ahp1 and the closely related phiKMV-like phages, suggesting that the Rz/Rz1 proteins are not used to enhance host lysis and alternative mechanisms may have evolved to enhance bacterial lysis.

### Phylogenetic relatedness of Ahp1

**Fig 6:**
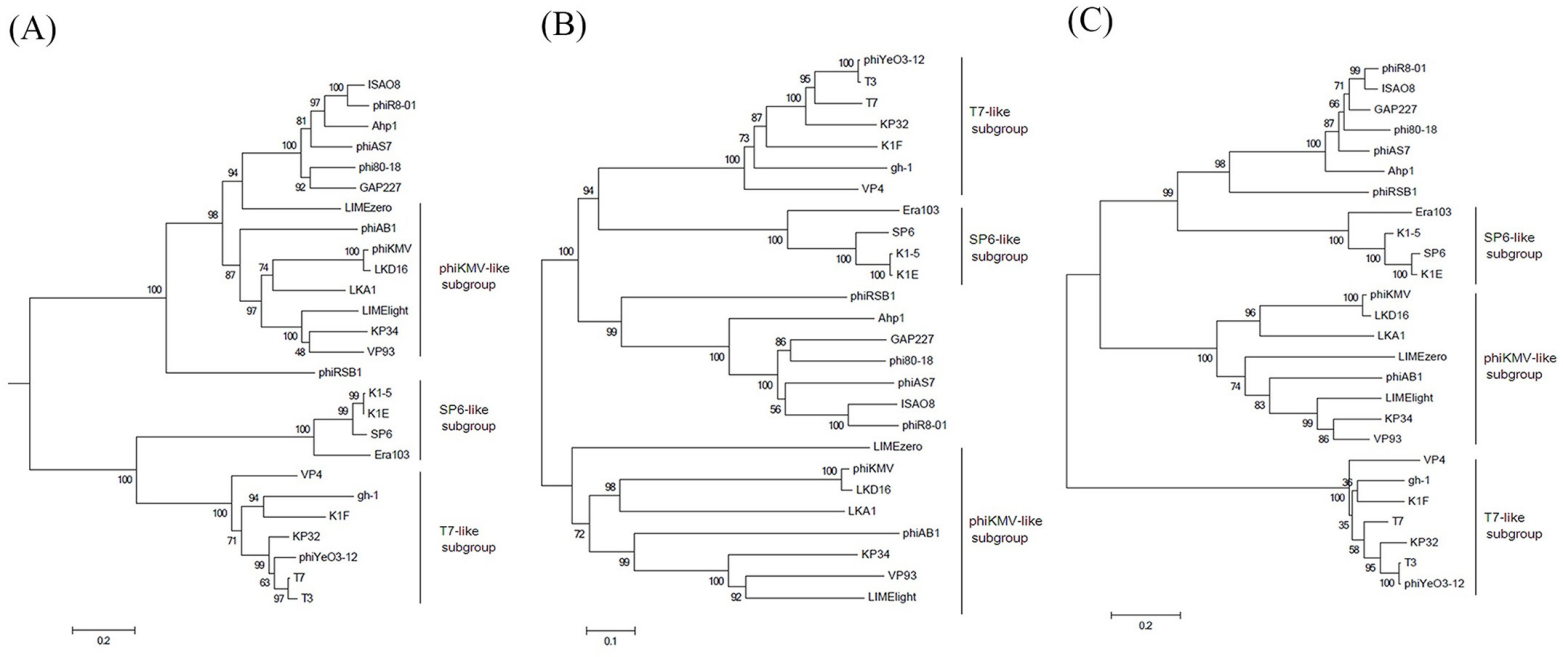
Phylogenetic relatedness among DNAP (A), RNAP (B), and major capsid proteins (C) from Ahp1 and some *Autographivirinae* phages based on amino acid sequence. The tree was drawn based on the neighbor joining algorithm using 1,000 bootstrap replicates, calculated from alignment results of MEGA program (version 6.0.6). Names of phages are shown on the right side.

As mentioned above, our data of ORF comparison suggested that phiKMV-like phages can be divided into at least two subgroups. To understand the relatedness between Ahp1 and the phiKMV-like phages, phylogenetic analysis was performed using DNAP, RNAP, and major capsid protein of Ahp1 (ORF20, ORF28, and ORF34, respectively) as the sample proteins. The proteins from *Autographivirinae* subfamily phages including T7-like phages, SP6-like phages, and phiKMV-like phages, each of which encodes its own single-subunit RNA polymerase [61] were also included. As shown in Fig 6, the proteins from Ahp1 were each clustered together with that of phiAS7, phi80-18, GAP227, phiR8-01, and ISAO8 and formed a clade distinct from those of T7-like, SP6-like, and the other phiKMV-like phages, which includes phiKMV and 7 related phages. Taken these together with the results of genomic comparison, we propose to classify Ahp1, phiAS7, phi80-18, GAP227, phiR8-01, and ISAO8 into a new subgroup, designated as Ahp1-like subgroup, within the *Autographivirinae* subfamily.

## Conclusions

In this study, a novel podophage of *A*. *hydrophila*, designated Ahp1, has been isolated and characterized. Phylogenetic relatedness among DNAP, RNAP, and major capsid protein suggest that a new subgroup, designated Ahp1-like subgroup, has formed within the *Autographivirinae*, in addition to T7-like, SP6-like, and phiKMV-like subgroups. Since Ahp1 has a narrow host range, for effective phage therapy, different phages are needed for preparation of effective cocktails that are capable of killing the heterogeneous *A*. *hydrophila* strains.

## Supporting Information

S1 Fig Adsorption of Ahp1 to its host *A*. *hydrophila* ATCC 7966. Unadsorbed phage in supernatants as assayed. Values are means of three independent experiments which exhibited negligible variations for the same time points.
(TIF)

S2 Fig One-step growth of Ahp1 on *A*. *hydrophila* strain ATCC 7966. Values are means of three independent experiments. Symbols: L, latent period; B, burst size.
(TIF)

S3 Fig Genome organization of Ahp1 and *Aeromonas salmonicida* phage phiAS7. Predicted ORFs are numbered for Ahp1and phiAS7. The ruler below represents the features of the genome.
(TIF)
