# Supplementary figures and images for: Genomic Characterization of the Novel Aeromonas hydrophila Phage Ahp1 Suggests the Derivation of a New Subgroup from phiKMV-Like Family
Source: PLoS One. 2016 Sep 7;11(9):e0162060. doi: 10.1371/journal.pone.0162060 (PMC5014404; doi:10.1371/journal.pone.0162060)

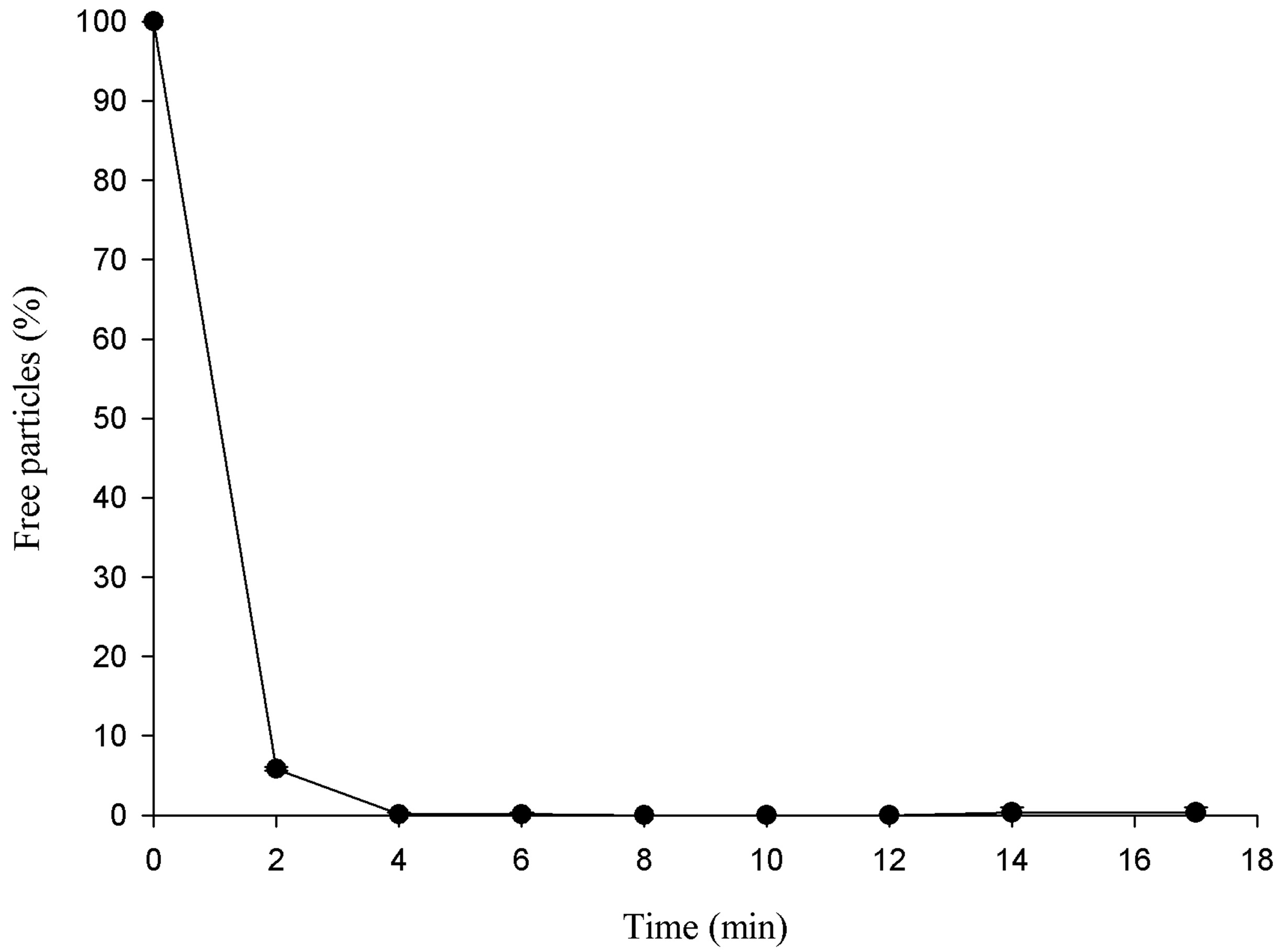

Supplement: S1 Fig — Unadsorbed phage in supernatants as assayed. Values are means of three independent experiments which exhibited negligible variations for the same time points. (TIF) [file pone.0162060.s001.tif]

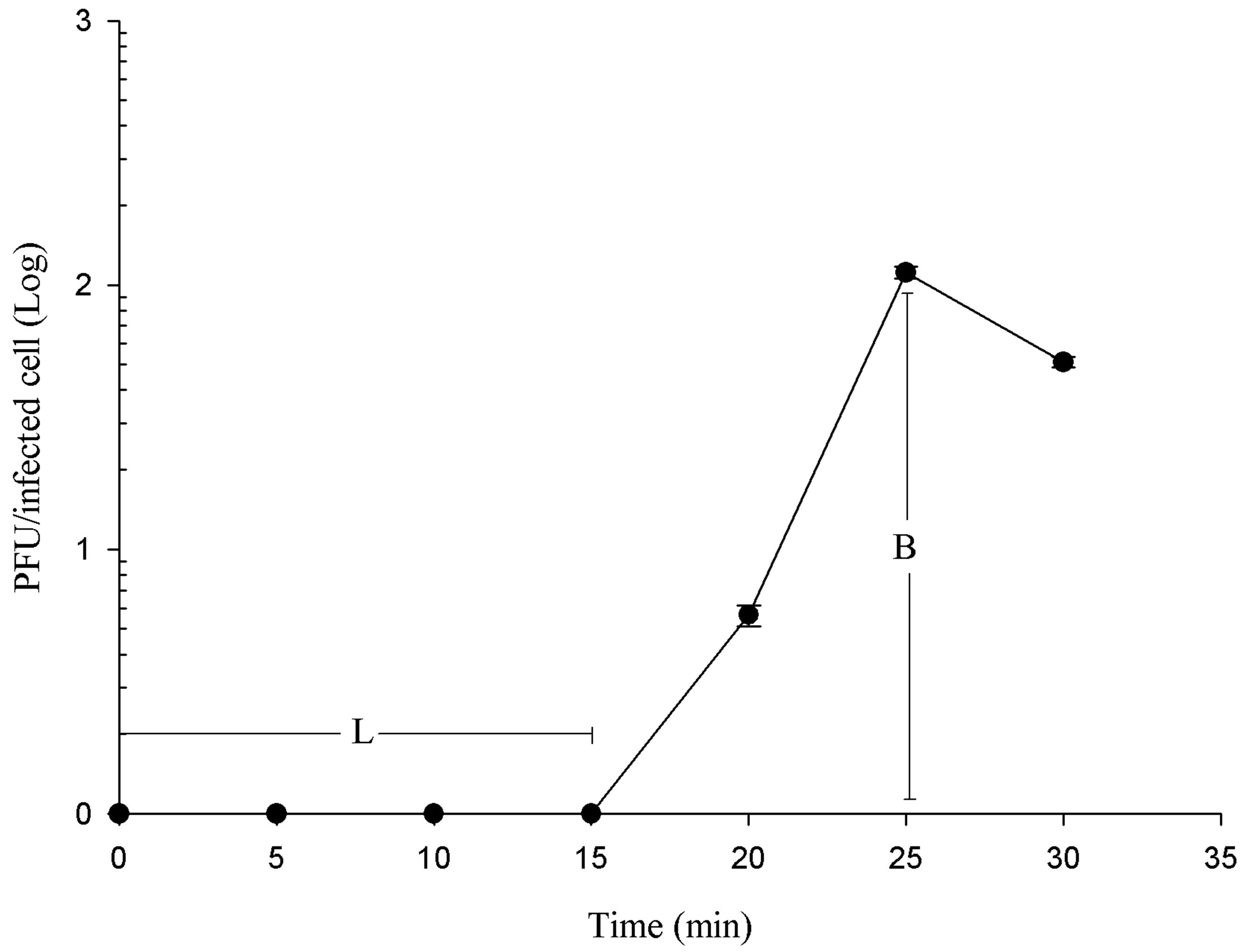

Supplement: S2 Fig — Values are means of three independent experiments. Symbols: L, latent period; B, burst size. (TIF) [file pone.0162060.s002.tif]

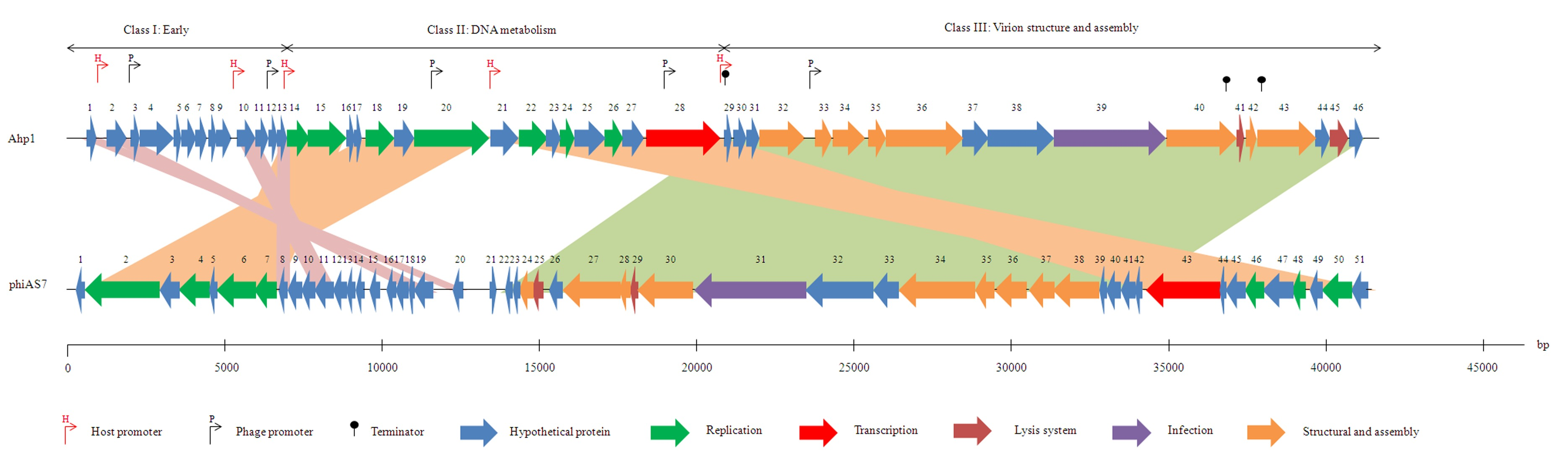

Supplement: S3 Fig — Predicted ORFs are numbered for Ahp1and phiAS7. The ruler below represents the features of the genome. (TIF) [file pone.0162060.s003.tif]
